# Malignant B Cells Induce the Conversion of CD4^+^CD25^−^ T Cells to Regulatory T Cells in B-Cell Non-Hodgkin Lymphoma

**DOI:** 10.1371/journal.pone.0028649

**Published:** 2011-12-09

**Authors:** Yixiang Han, Jianbo Wu, Laixi Bi, Shudao Xiong, Shenmeng Gao, Lihui Yin, Lei Jiang, Chiqi Chen, Kang Yu, Shenghui Zhang

**Affiliations:** 1 Laboratory of internal medicine, the First Affiliated Hospital of Wenzhou Medical College, Wenzhou, China; 2 Department of Hematology, the First Affiliated Hospital of Wenzhou Medical College, Wenzhou, China; 3 Department of Hematology/Oncology, the Second Hospital of Anhui Medical University, Hefei, China; 4 Key Laboratory of Molecular Medicine, Ministry of Education, and Department of Biochemistry and Molecular Biology, Fudan University Shanghai Medical College, Shanghai, China; The University of Chicago, United States of America

## Abstract

Recent evidence has demonstrated that regulatory T cells (Treg) were enriched in the tumor sites of patients with B-cell non-Hodgkin lymphoma (NHL). However, the causes of enrichment and suppressive mechanisms need to be further elucidated. Here we demonstrated that CD4^+^CD25^+^FoxP3^+^CD127^lo^ Treg were markedly increased and their phenotypes were different in peripheral blood (PB) as well as bone marrow (BM) from newly diagnosed patients with B-cell NHL compared with those from healthy volunteers (HVs). Involved lymphatic tissues also showed higher frequencies of Treg than benign lymph nodes. Moreover, the frequencies of Treg were significantly higher in involved lymphatic tissues than those from PB as well as BM in the same patients. Suppression mediated by CD4^+^CD25^+^ Treg co-cultured with allogeneic CFSE-labeled CD4^+^CD25^−^ responder cells was also higher in involved lymphatic tissues from B-cell NHL than that mediated by Treg from HVs. In addition, we found that malignant B cells significantly induced FoxP3 expression and regulatory function in CD4^+^CD25^−^ T cells in vitro. In contrast, normal B cells could not induce the conversion of CD4^+^CD25^−^ T cells to Treg. We also showed that the PD-1/B7-H1 pathway might play an important role in Treg induction. Taken together, our results suggest that malignant B cells induce the conversion of CD4^+^CD25^−^ T cells to Treg, which may play a role in the pathogenesis of B-cell NHL and represent a promising therapeutic target.

## Introduction

B-cell non-Hodgkin lymphomas (NHL) are common lymphoid malignancies in which malignant B cells arrested at varying stages of differentiation often involve lymph nodes (LNs) and occasionally extranodal tissues. Besides malignant cells, T cells are usually present in the tumor microenvironment, which commonly include regulatory T cells (Treg) [1], suggesting that Treg may play a role in regulating tumor growth in patients with B-cell NHL.

It is becoming clear that tumors may induce immunologic tolerance by promoting the expansion, recruitment and activation of Treg [2]. Treg, which account for about 5% to 10% of peripheral CD4^+^ T cells in both mice and humans, include naturally occurring CD4^+^CD25^+^ Treg as well as peripherally induced CD4^+^ Treg [3], [4]. Recently, Helios, a member of the Ikaros family, was reported as a marker to express on the naturally occurring Treg, but not those induced Treg [5]. Sustained high surface expression of CD25, cytotoxic T-lymphocyte–associated antigen 4 (CTLA-4) and glucocorticoid-induced TNFR-related protein (GITR) expression are features of suppressive Treg [6], [7]. Expression of forkhead box P3 (FoxP3), a member of the forkhead-winged helix family of transcription factor, has been demonstrated to be a lineage marker for murine and human Treg [7], [8]. Furthermore, they have been found that Treg express lower levels of CD127 than the majority of other CD4^+^ T cells, and CD127 expression inversely correlates with FoxP3 expression in our own and others' previous studies [9], [10], [11]. Thus, the gate of CD4^+^CD25^+^FoxP3^+^CD127^lo^ is currently one of the best methods to define for Treg using flow cytometry [12], [13].

Treg have been shown to be overrepresented in patients with various cancers and able to suppress antitumor immune response [14], [15], [16], [17]. In B-cell NHL, several studies have also shown that increased frequencies of CD4^+^CD25^+^ Treg in peripheral blood (PB) and involved LNs mediated vigorous suppressive activity compared to those from healthy volunteers (HVs) [18], [19]. Although many studies have shown that Treg play an importance role in the immune system, the exact mechanism of suppression by Treg is still poorly understood. It has been shown that Treg can be efficiently recruited and expanded in the tumor microenvironment and suppress effector function of cytotoxic T cells as well as NK cell-mediated cytotoxicity [20], [21], [22], [23], [24]. Several studies have already demonstrated that Treg cell-mediated immunosuppression is one of the crucial tumor immune evasion mechanisms and the main obstacles of successful cancer immunotherapy [11], [25]. These studies suggest that Treg could be an attractive target for anti-cancer therapy in both humans and animal models.

In some kinds of B-cell NHL, such as mantle cell lymphoma (MCL), malignant B cells usually infiltrate other parts of the body including bone marrow (BM) when they are diagnosed. Here we measured the frequencies and suppressive function of Treg in PB, BM and involved lymphatic tissues of patients with B-cell NHL and determined whether malignant B cells could induce the conversion of Treg from CD4^+^CD25^−^ T cells. We found that the elevated frequencies and suppressive function of Treg might be related to the pathogenesis of B-cell NHL, and the conversion of Treg was induced by malignant B cells through the PD-1/B7-H1 pathway in some kinds of B-cell NHL, especially in MCL.

## Materials and Methods

### Patients

Between September 2008 and March 2011, 32 newly diagnosed patients with B-cell NHL (age range: 33 to 81 years, mean = 59, SD = 14) were recruited from the First Affiliated Hospital of Wenzhou Medical College. All protocols and experiments were approved by the First Affiliated Hospital of Wenzhou Medical College institutional review board for clinical experiments and use of human samples; written consents were obtained from all subjects participated in this study in accordance with the Declaration of Helsinki protocol. The age-matched control group comprised of 18 HVs (age range: 35 to 72 years, mean = 55, SD = 13) and 4 individuals with benign lymphadenopathy (age range: 41 to 65 years, mean = 52, SD = 10). Patient characteristics are described in Table 1. Although various classification systems exist for determining specific histological subtypes, B-cell NHL is usually divided into indolent and aggressive forms. So we divided these patients into tow groups: indolent and aggressive.

**Table 1:** Clinical characteristics of patients with B-cell NHL.

Patient no.	Sex	Age,y	Histologic diagnosis	Tissue used
1	M	47	DLBCL	PBMC, BM
2	M	72	MCL	PBMC, BM (new)
3	M	43	MCL	PBMC, BM (new)
4	F	46	DLBCL	PBMC, Node (new)
5	M	57	MCL	PBMC, BM
6	M	65	MZL	PBMC, Node (new)
7	F	65	SLL	PBMC, BM, Node (new)
8	M	70	DLBCL	PBMC, BM
9	M	53	SLL	PBMC, BM (new)
10	M	76	SLL	PBMC, BM (new)
11	M	63	MCL	PBMC, BM (new)
12	M	36	DLBCL	PBMC, BM
13	M	33	DLBCL	PBMC, BM, Node (new)
14	F	58	DLBCL	PBMC, BM (new)
15	M	60	MZL	PBMC, BM, Node (new)
16	M	49	Follicular NHL, grade 1	PBMC, Node (new)
17	F	44	DLBCL	PBMC, BM
18	F	56	DLBCL	PBMC, BM, Spleen (new)
19	M	78	MCL	PBMC, BM
20	M	78	DLBCL	PBMC, BM, Node (new)
21	M	35	MZL	PBMC, BM (new)
22	M	54	DLBCL	PBMC, BM
23	M	71	DLBCL	PBMC, BM (new)
24	M	49	DLBCL	PBMC, BM, Node (new)
25	F	66	DLBCL	PBMC, BM (new)
26	F	73	DLBCL	PBMC, BM
27	M	53	MCL	PBMC, BM (new)
28	M	33	DLBCL	PBMC, BM
29	M	57	DLBCL	PBMC, Node (new)
30	F	73	DLBCL	PBMC, BM (new)
31	F	80	DLBCL	PBMC, BM
32	M	81	DLBCL	PBMC, Node (new)

DLBCL indicates diffuse large B-cell lymphoma; MCL, mantle cell lymphoma; new, a diagnosis of lymphoma with no previous history of lymphoproliferative disease; MZL, marginal zone lymphoma; and SLL, small lymphocytic lymphoma.

### Reagents

The following fluorochrome-conjugated antibodies were used for immunostaining: anti-CD25-FITC, anti-CD25-PE, anti-CD4-PerCP, anti-FoxP3-PE, anti-FoxP3-Alexa Fluor® 647, anti-CD127-Alexa Fluor® 647, anti-CD45RA-PE, anti-CD45RO-PE, anti-HLA-DR-FITC, anti-CD62L-PE, anti-CD95-PE, anti-CTLA-4-PE, anti-PD-1-PE, anti-CD3-PerCP, anti-CD8-FITC, anti-IFN-γ-PE, anti-IL-4-PE, anti-IL-2-PE, anti-IL-17-PE, anti-CD19-FITC, anti-CD19-APC, anti-Lambda-FITC, anti-Kappa-PE, anti-B7-H1-PE and appropriate negative controls all from BD (San Diego, CA). Anti-GITR-FITC and recombinant human PD-1 Fc were obtained from R&D systems (Minneapolis, MN). CD4^+^CD25^+^ Regulatory T-cell Isolation Kit, B Cell Isolation Kit II and magnetic-activated cell sorting columns were purchased from Miltenyi Biotec (Bergisch Gladbach, Germany). Carboxyfluorescein diacetate succinimidylester (CFSE) was obtained from Alexis (Lausen, Switzerland). Phorbol 12-myristate 13-acetate (PMA), ionomycin and brefeldine A (BFA) were purchased from Sigma-Aldrich (St Louis, MO).

### Immunostaining and flow cytometric analysis

Fresh lymphatic tissue specimen was cut into approximately 1 mm pieces and disaggregated mechanically into a single cell suspension by rotating knifes (Medimachine; Dako, Hamburg, Germany). The cell suspension was subsequently centrifuged through Ficoll-Hypaque to isolate mononuclear cells. Peripheral blood mononuclear cells (PBMCs) and bone marrow mononuclear cells (BMMCs) were isolated from PB and BM samples by Ficoll-Hypaque centrifugation, respectively. Cells were incubated with fluorochrome-conjugated antibodies which recognize and bind to various cell surface antigens. For intracellular FoxP3 staining, cells were first stained with appropriate surface antibodies, washed, and then fixed, permeablized and stained with anti-FoxP3 for 30 min at 4°C in the dark. For intracellular cytokine staining, cells were stimulated with PMA (20 ng/mL) and ionomycin (1 µg/mL) for 5 hours in the presence of BFA (10 µg/mL). Samples were subsequently incubated with anti-CD3-PerCP and anti-CD8-FITC. Then, the cells were fixed, permeabilized and stained with anti-FoxP3-Alexa Fluor® 647, anti-IFN-γ-PE, anti-IL-4-PE, anti-IL-2-PE, and anti-IL-17-PE. A minimum of 50,000 cells were analyzed for each sample using flow cytometry (FACSCalibur; BD).

### Suppression assay

CD4^+^CD25^+^ and CD4^+^CD25^−^ T cells were obtained using CD4^+^CD25^+^ Regulatory T cell Isolation Kit according to manufacturer's protocols. The suppressive function of CD4^+^CD25^+^ Treg was assessed using mixed leukocyte culture assay. Briefly, CD4^+^CD25^+^ T cells were co-cultured with allogeneic CFSE (5 µmol/L)-labeled CD4^+^CD25^−^ T cells in the presence of γ-irradiated (30 Gy) PBMCs (as antigen-presenting cells) for 4 days, with each population 2×10^5^ cells. After 4 days of co-culture, the cells were washed and the CFSE intensity was analyzed by flow cytometry.

### Induction assay

CD19^+^ cells from PBMCs, BMMCs and involved lymphatic tissues were isolated by using B Cell Isolation Kit II according to manufacturer's instructions. CD4^+^CD25^−^ and CD19^+^ cells were co-cultured in 96-well flat-bottom plate at a density of 2×10^5^ cells/well for each cell type. These cells were cultured in X-ViVO™ 15 medium (Cambrex, Walkersville, MD) supplemented with 2 mM L-glutamine, 100 IU/ml IL-2 and 10% BSA in the presence or absence of human PD-1 Fc (20 µg/ml) or anti-human B7-H1 (10 µg/ml; eBioscience, San Diego, CA). After 5 days, cultured cells were harvested and stained for CD4 and intracellular FoxP3, and analyzed by flow cytometry.

### Statistical analysis

Quantitative data were expressed as mean ± standard deviation (SD). The frequencies of Treg for statistical differences in PB, BM and involved lymphatic tissues were analyzed with a one-way analysis of variance (ANOVA). Data about the conversion of Treg through PD-1/B7-H1 pathway were analyzed using pair t-test. A *p* value of less than 0.05 was considered significant.

## Results

### Elevated frequencies of CD4^+^CD25^+^FoxP3^+^CD127^lo^ Treg in patients with B-cell NHL

**Figure 1:**
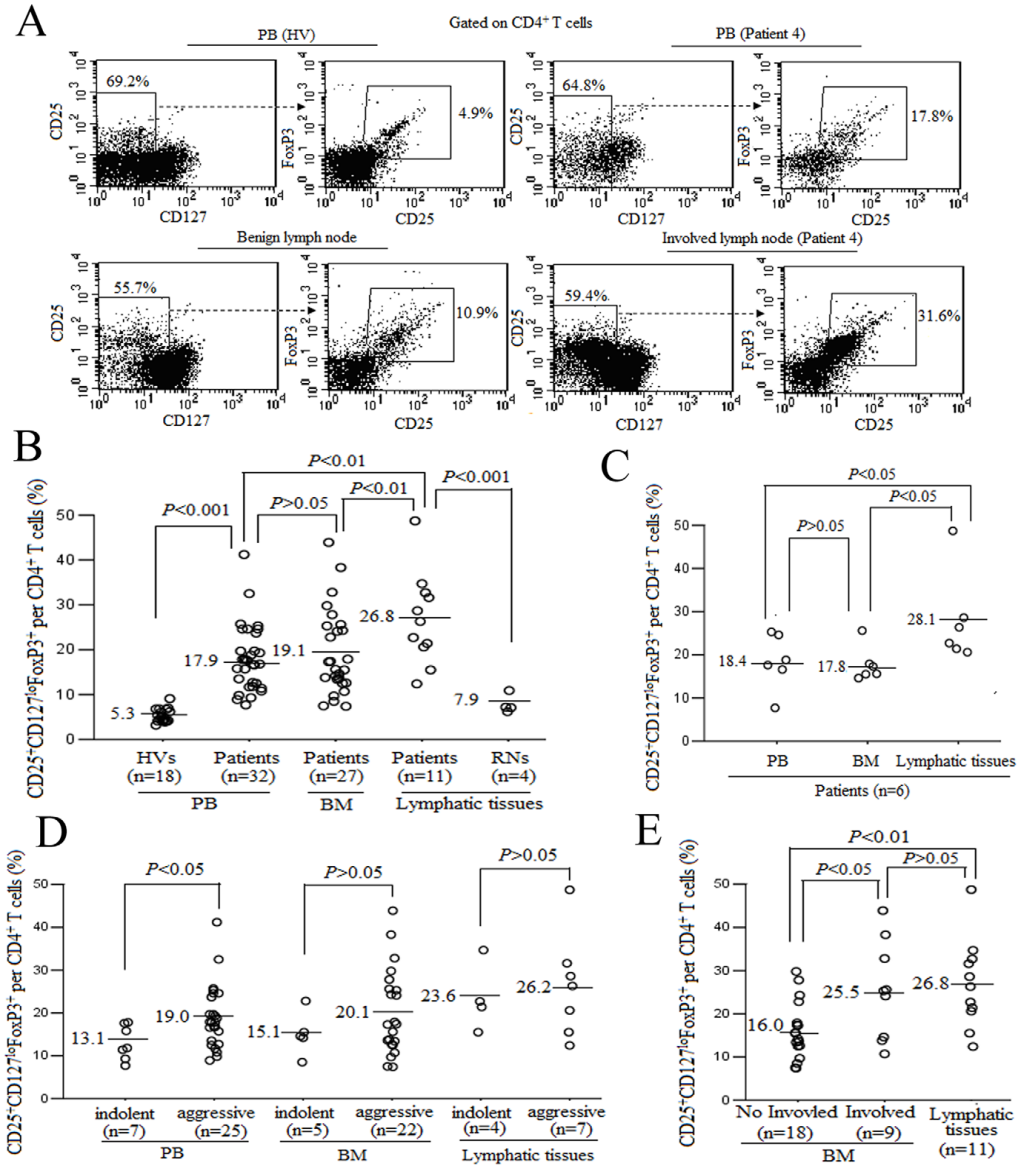
Elevated frequencies of CD4^+^CD25^+^FoxP3^+^CD127^lo^ Treg in PB, BM and involved lymphatic tissues in patients with B-cell NHL. (A) Dot plots were gated on CD4^+^ T cells (based on forward and side scatter and CD4 staining). The number in the dot plot represents the percentage of gated cells expressing the relevant marker. (B) Frequencies of CD4^+^CD25^+^FoxP3^+^CD127^lo^ Treg in PB, BM and involved lymphatic tissues from patients with B-cell NHL and HVs were quantified using flow cytometric analysis. (C) Frequencies of CD4^+^CD25^+^FoxP3^+^CD127^lo^ Treg in PB, BM and involved lymphatic tissues from the same patients with B-cell NHL were measured as described earlier. (D) Frequencies of CD4^+^CD25^+^FoxP3^+^CD127^lo^ Treg in PB, BM and involved lymphatic tissues from indolent B-cell NHL patients and aggressive B-cell NHL patients were measured as described earlier. (E) Frequencies of CD4^+^CD25^+^FoxP3^+^CD127^lo^ Treg in BM and involved lymphatic tissues from patients with B-cell NHL were measured as described earlier. Each open circle represents a single individual assessed in the respective group and numbers on the left of horizontal bars represent the group means.

To investigate a possible role of CD4^+^CD25^+^FoxP3^+^CD127^lo^ Treg in the immune system in B-cell NHL, both peripheral tolerance and the tumor microenvironment, we measured the frequencies of CD4^+^CD25^+^FoxP3^+^CD127^lo^ Treg in PB, BM and involved lymphatic tissues from patients with B-cell NHL. For comparison, PBMCs from HVs and cells from benign LNs were used as controls. As shown in Figure 1A and 1B, the elevated frequencies of CD4^+^CD25^+^FoxP3^+^CD127^lo^ Treg could be detected in involved lymphatic tissues (26.8%±10.1% of the total CD4^+^ T cells, range, 12.4%–48.7%, n = 11) and PB (17.9%±7.3%, range, 7.7%–41.2%, n = 32) and BM (19.1%±9.4%, range, 7.4%–43.9%, n = 27) compared with those from PBMCs from HVs (5.3%±1.5%, range, 3.2%–9.1%, n = 18) or benign LNs (7.9%±2.1%, range, 6.2%–10.9%, n = 4). The Treg frequencies in involved lymphatic tissues (28.1%±10.6%, range, 20.6%–40.7%) were higher compared to PB (18.4%±6.4%, range, 7.7%–25.3%) or BM (17.8%±4.0%, range, 14.6%–25.6%) in the same patients (n = 6, Figure 1C), indicating that preferential accumulation of Treg in involved lymphatic tissues occurred. Furthermore, patients with aggressive B-cell NHL (n = 25; DLBCL, n = 19; and MCL, n = 6) had higher frequencies of Treg in PB than patients with indolent B-cell NHL (n = 7; MZL, n = 3; and SLL, n = 3; and FL, n = 1). However, there was no significant difference in BM or involved lymphatic tissues between indolent and aggressive B-cell NHL (Figure 1D). As this is generally true for B-cell NHL which may have BM involvement of malignant B cells, we analyzed the influences of BM involvement on the Treg frequencies. Patients with BM involvement (n = 9; MCL, n = 5; and DLBCL, n = 3; and SLL, n = 1) had higher frequencies of Treg in BM than patients without BM involvement (n = 18). Moreover, there was no significant difference in involved BM and involved lymphatic tissues (Figure 1E).

### Surface phenotype of CD4^+^CD25^+^CD127^lo^ Treg

**Figure 2:**
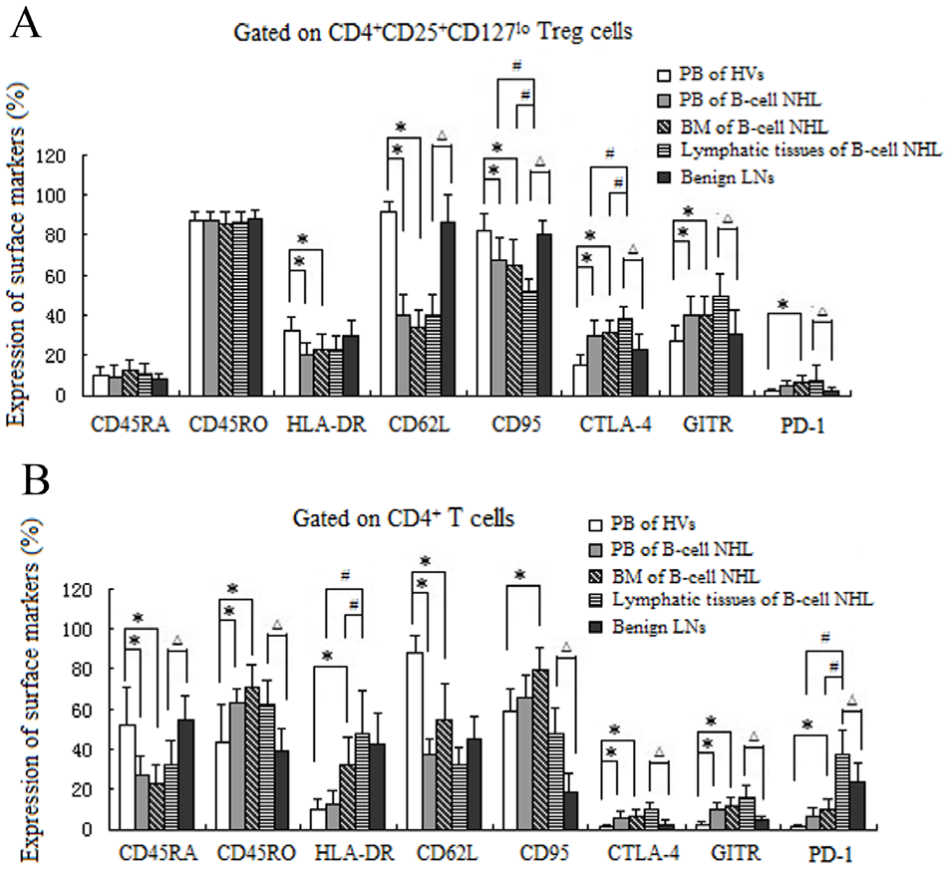
Surface phenotype of CD4^+^CD25^+^CD127^lo^ Treg and CD4^+^ T cells obtained from patients with B-cell NHL and HVs. Expression rates of various markers in PB of HVs (n = 8), PB (n = 8; patients 3, 4, 7, 8, 11, 13, 15, and 16), BM (n = 8; patients 3, 8, 11, 12, 13, 17, 19, and 20)and involved lymphatic tissues (n = 8; patients 4, 7, 13, 15, 16, 18, 20, and 24) from patients with B-cell NHL (n = 8), and benign LNs (n = 4). (A). CD4^+^CD25^+^CD127^lo^ Treg. (B). CD4^+^ T cells. ^*^ *P*<0.05 vs Treg or CD4^+^ T cells from PB of HVs; ^Δ^ *P*<0.05 vs Treg or CD4^+^ T cells from benign LNs; ^#^ *P*<0.05 vs Treg or CD4^+^ T cells from involved lymphatic tissues of B-cell NHL.

As the suppressive function of Treg is associated with its surface receptors [6], [26], [27], we evaluated a possible role of surface markers in CD4^+^CD25^+^CD127^lo^ Treg and CD4^+^ T cells presented in patients with B-cell NHL. As shown in Figure 2A and 2B, significant differences in phenotypes were found between patients with B-cell NHL and HVs. The CD4^+^CD25^+^CD127^lo^ Treg had high expression of CD45RO combined with low expression of CD45RA and no difference in the expression of CD45RA and CD45RO between patients with B-cell NHL and HVs. The expression levels of HLA-DR, CD62L and CD95 were reduced in Treg of PB, BM and involved lymphatic tissues from patients with B-cell NHL compared with those from PB samples of HVs and benign LNs. In contrast, the expression levels of CTLA-4 and GITR in Treg of PB, BM and involved lymphatic tissues from B-cell NHL were significantly higher than those in PB samples from HVs and benign LNs. Furthermore, the expression levels of CTLA-4 and GITR in Treg from involved lymphatic tissues were slightly higher than those from PB or BM in patients with B-cell NHL. Similar expression levels of CTLA-4 and GITR occurred in both PB and BM samples. The expression of PD-1 was higher in Treg from BM samples of patients with B-cell NHL than those from HVs. In addition, the expression of PD-1 in Treg was also higher in involved lymphatic tissues from patients with B-cell NHL than those from benign LNs. Notably, the infiltrating CD4^+^ T cells expressed high levels of PD-1 in the involved lymphatic tissues, indicating that PD-1 was mainly expressed on the CD4^+^CD25^−^ cells, but not on Treg.

### Functional characterization of CD4^+^CD25^+^FoxP3^+^CD127^lo^ Treg from patients with B-cell NHL

**Figure 3:**
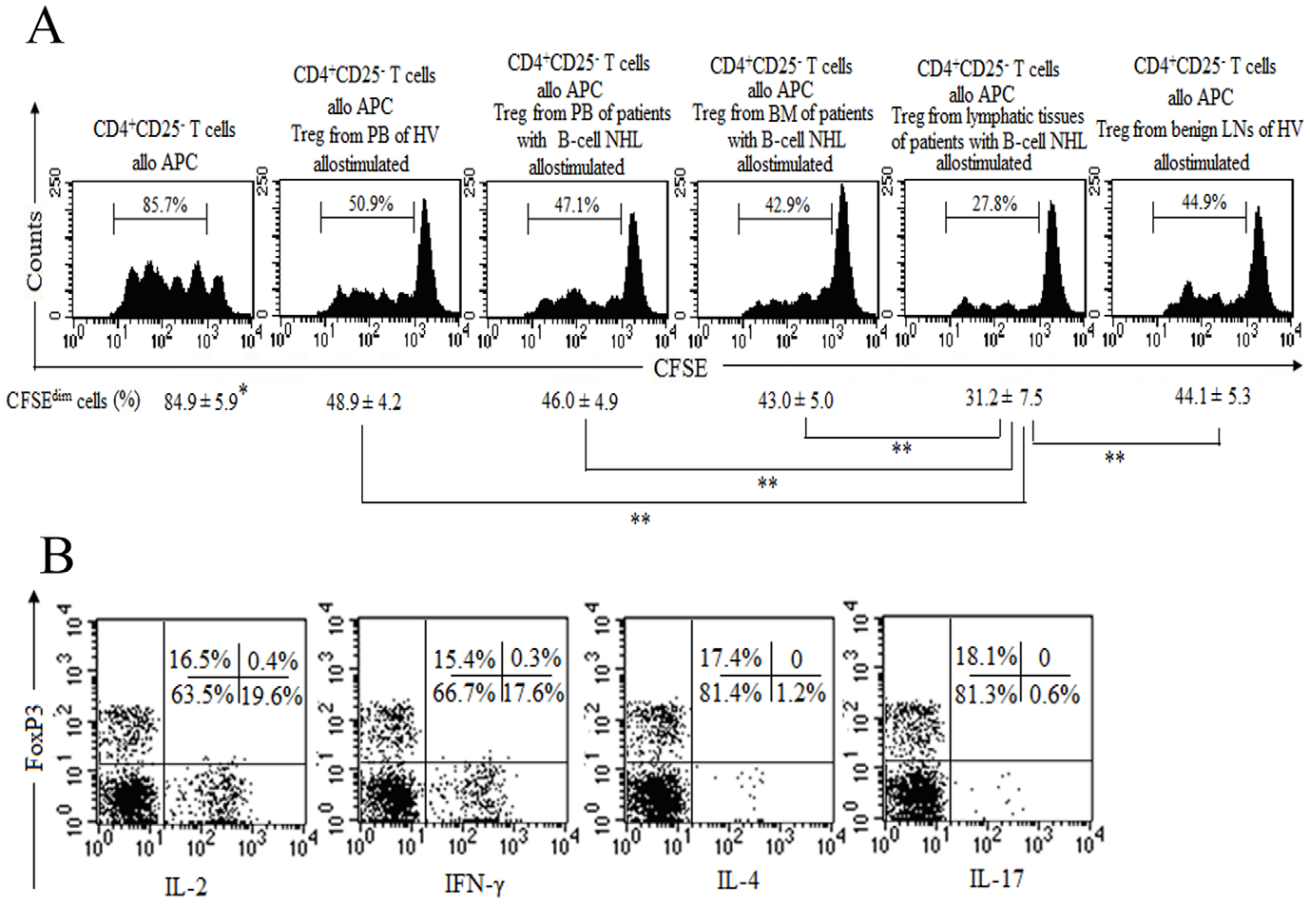
Functional analysis of Treg from patients with B-cell NHL in vitro. (A) Fresh isolated Treg (CD4^+^CD25^+^ T cells) were cultured for 48 hours and then cocultured with CFSE-labeled CD4^+^CD25^−^ T cells for 4 days in the presence of γ-irradiated (30 Gy) PBMCs, with each population 2×10^5^ cells. Proliferation was measured by levels of CFSE dilution using flow cytometric analysis. Histograms show the profile of CFSE-labeled CD4^+^CD25^−^ T cells and the proliferated CD4^+^CD25^−^ T cells was measured by the percentage of CFSE^dim^ cells, as indicated. Treg could inhibit the proliferation of allogeneic CD4^+^CD25^−^ T cells (^*^ *P*<0.01 vs CD4^+^CD25^−^ T cells only). The involved lymphatic tissues were reported as positive by pathology in the same lymph nodes or spleen. The suppressive activity of Treg from involved lymphatic tissues was higher than those from PB or BM in the same patients as well as those from HVs (^**^ *P*<0.01). One representative experiment of four was presented. (B) Involved lymphatic tissues from patients with B-cell NHL were disaggregated into a single cell suspension and stimulated with PMA plus ionomycin for 5 hours in the presence of BFA. Cells were subsequently costained with anti-CD3 and anti-CD8. Next, cells were fixed, permeabilized and stained with anti-FoxP3, anti-IL-2, anti-IFN-γ, anti-IL-4 or anti-IL-17. Dot plots were gated on CD3^+^CD8^−^ (CD3^+^CD4^+^) T cells, and the numbers within each quadrant represent the percentages of CD4^+^ T cells. The results suggested that Treg of involved lymphatic tissues from patients with B-cell NHL could not produce and secret IL-2, IFN-γ, IL-4 and IL-17. A representative of four independent experiments was showed.

As the functional hallmark of Treg is their ability to suppress immune responses driven by effector T cells, we performed a typical in vitro suppression assay to examine the suppressive capacity of these cells. The CD4^+^CD25^+^ T cells used in these experiments were indeed confirmed FoxP3^+^ in equal proportions and not contaminated by activated non-Treg CD4^+^ T cells. CFSE-labeled CD4^+^CD25^−^ T cells (responding cells) were co-cultured with CD4^+^CD25^+^ T cells (stimulating cells) in the presence or absence of γ-irradiated PBMCs for 4 days. At a suppressor/ responder ratio of 1∶1, the suppression of responder cell proliferation was higher in involved lymphatic tissues from patients with B-cell NHL (n = 4; patients 6, 7, 13, and 18)than PB or BM samples from patients with B-cell NHL (patients 6, 7, 13, and 18 for PB, n = 4; and patients 7, 11, 13, and 18 for BM, n = 4) as well as PB samples from HVs or benign LNs (Figure 3A). In addition, Treg, irrespective of the source, could markedly inhibit the proliferation of CD4^+^CD25^−^ T cells (Figure 3A).

Another inherent attribute of Treg is their disability to produce and secrete proinflammatory cytokines such as IL-2, IFN-γ, IL-4 and IL-17. We assessed the production of these cytokines in CD4^+^FoxP3^+^ T cell subset using intracellular cytokine staining after stimulation with PMA/ionomycin. As showed in Figure 3B, CD4^+^FoxP3^+^ T cells from involved lymphatic tissues of patients with B-cell NHL (n = 4; patients 6, 7, 13, and 18) were unable to produce IL-2, IFN-γ, IL-4 and IL-17 in response to strong PMA/ionomycin stimulation. Similarly, CD4^+^FoxP3^+^ T cells from PB of HVs could not produce these cytokines (data not shown). These results indicated that both the inhibitory immune response and produced cytokine profiles accorded to typical characteristics of human Treg.

### Malignant B cells induce CD4^+^CD25^−^ T cells into Treg in vitro

**Figure 4:**
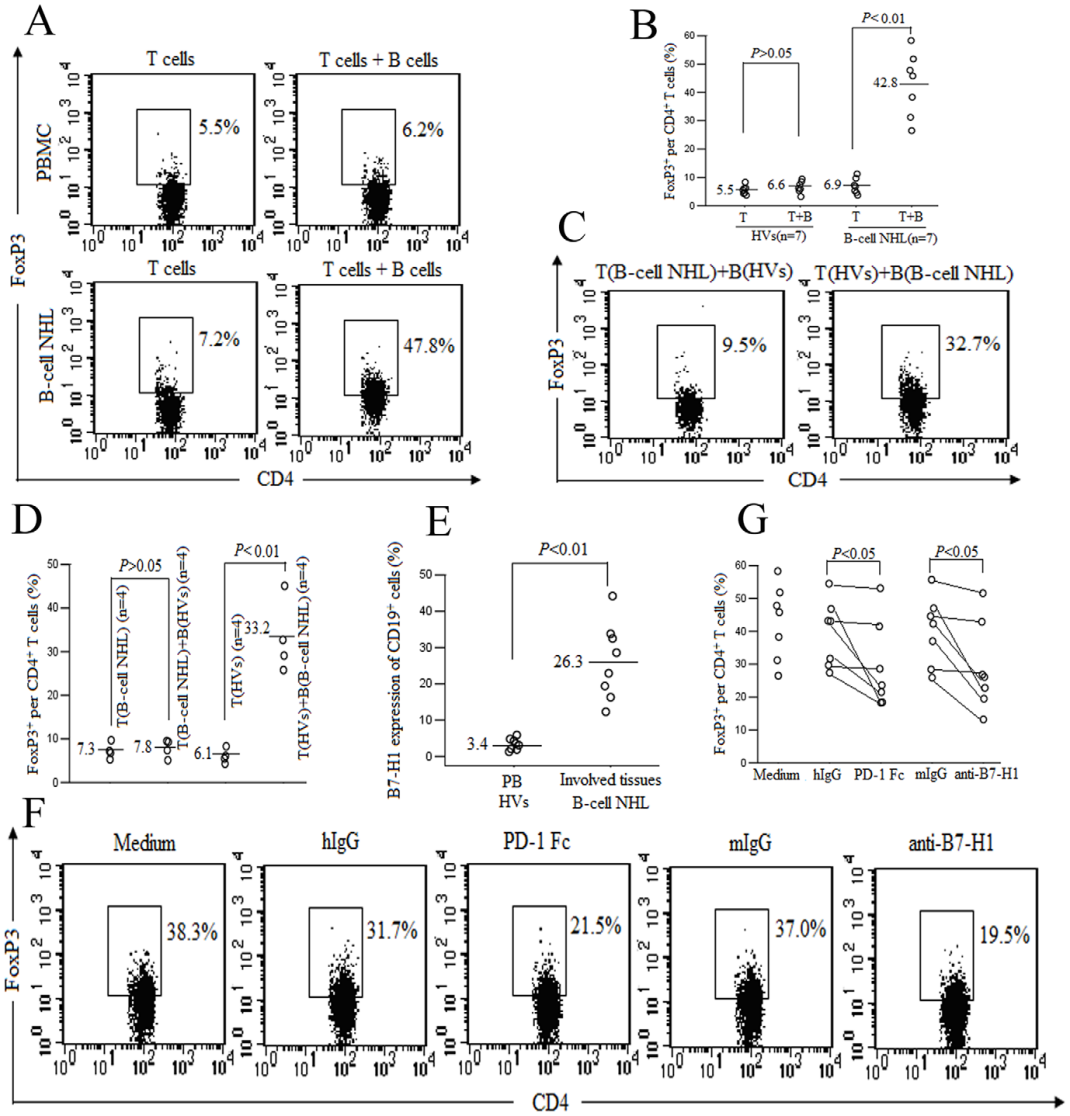
Malignant B cells induce FoxP3 expression and Treg conversion from CD4^+^CD25^−^ T cells, which partly depends on the interaction of between PD-1 and B7-H1. (A,B) Malignant B cells, but not normal B cells, were able to induce FoxP3 expression in autologous conventional T cells. CD4^+^CD25^−^ T cells were cultured for 5 days with X-ViVO™ 15 medium alone or purified autologous B cells in the presence of 100 IU/ml IL-2. The cells were then harvested, stained, and analyzed for the percentage of CD4^+^FoxP3^+^ cells. The number in the dot plot represents the percentage of gated cells expressing the relevant marker. Each open circle represents a single individual and numbers on the left of horizontal bars represent the group means. The results were representative of seven independent experiments. (C,D) Malignant B cells, but not tumor-infiltrating T cells, were able to induce the conversion of Treg from conventional T cells. CD4^+^CD25^−^ T cells isolated from involved lymphatic tissues of B-cell NHL were co-cultured with normal B cells for 5 days. CD4^+^CD25^−^ T cells isolated from PB of HVs were co-cultured with malignant B cells for 5 days. (E) The expression of B7-H1 was higher in lymphoma B cells than normal B cells. Cells were costained with anti-CD19-FITC and anti-B7-H1-PE, and then were analyzed the expression of B7-H1 on CD19^+^ B cells using flow cytometry. (F,G) Histograms showed the effect of the interaction between PD-1 and B7-H1 on the conversion of Treg from conventional T cells. CD4^+^CD25^−^ T cells were cocultured with autologous B cells purified from patients with B-cell NHL for 5 days. Cells were treated with PD-1 fusion protein or anti-B7-H1 antibody as well as their corresponding controls in the coculture system. A CD4^+^FoxP3^+^ gate was drawn based on their isotype controls. The results were representative of seven independent experiments.

To investigate why the frequencies of Treg were increased in patients with B-cell NHL, sorted CD4^+^CD25^−^ cells from patient samples (patients 5, 8, 11, 19, and 27 for BM, n = 5; patients 7 and 24 for involved LN, n = 2) were co-cultured with purified autologous malignant B cells. The malignant B cells were verified by flow cytometry to be clonally restricted, more than 90% of cells expressed either kappa or lambda immunoglobulin, but not both. We found that FoxP3^+^ cells were markedly induced from CD4^+^CD25^−^ T cells in the presence of autologous malignant B cells. In addition, FoxP3^+^ cells could not be induced from CD4^+^CD25^−^ T cells in the presence of autologous normal B cells from HVs (Figure 4A and 4B). Furthermore, we found that FoxP3^+^ cells were also induced from CD4^+^CD25^−^ T cells isolated from HVs in the presence of malignant B cells, and FoxP3^+^ cells could not be induced from CD4^+^CD25^−^ T cells isolated from B-cell NHL specimens (patients 5 and 11 for BM, n = 2; patients 7 and 24 for involved LN, n = 2) in the presence of normal B cells (Figure 4C and 4D). Thus, these results indicated that the conversion of Treg was dependent on malignant B cells, irrespective of the origin of tissues, not T cells, which further confirmed and extended the previous conclusions by other researcher in involved LNs from FL [28].

B7-H1, a newly discovered member of the B7 family, is expressed on monocytes or dendritic cells. We showed that the expression of B7-H1 was higher in lymphoma B cells than normal B cells (Figure 4E). Surprising, in patients 5, 11, 19, and 27, which were MCL, blocking the interaction of PD-1 and B7-H1 using PD-1 Fc or anti-B7-H1 antibody could partly inhibit the induction of the CD4^+^FoxP3^+^ phenotype (Figure 4F). However, in patients 7, 8, and 24 (SLL, n = 1; and DLBCL, n = 2), blocking the interaction of PD-1 and B7-H1 could not inhibit the induction of the CD4^+^FoxP3^+^ phenotype. In a whole, blocking the PD-1/B7-H1 pathway could reduce the generation of Treg (Figure 4G), suggesting that PD-1/B7-H1 signaling may regulate the generation of Treg in B-cell NHL, especially in MCL.

**Figure 5:**
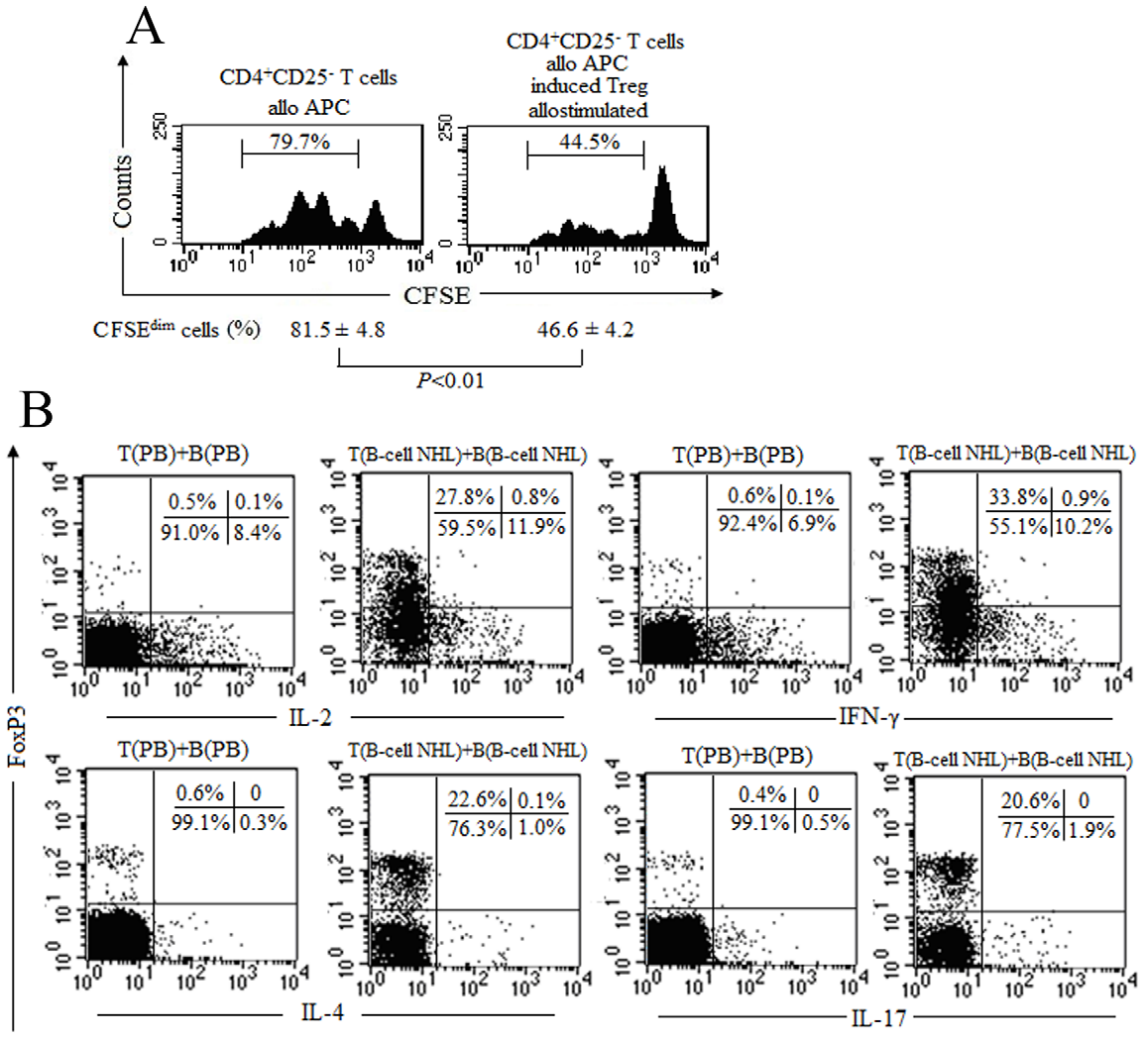
Functional analysis of induced FoxP3-expressing CD4 cells in vitro. (A) CD4^+^CD25^−^ cells isolated from a patient with B-cell NHL were co-cultured with purified autologous B cells for 5 days. Then, CD4^+^CD25^+^ T cells were isolated and cocultured with CFSE-labeled CD4^+^CD25^−^ T cells for 4 days in the presence of γ-irradiated (30 Gy) PBMCs, with each population 2×10^5^ cells. Histograms showed the profile of CFSE-labeled CD4^+^CD25^−^ T cells and the proliferated CD4^+^CD25^−^ T cells was measured by the percentage of CFSE^dim^ cells, as indicated. The converted Treg could inhibit the proliferation of allogeneic CD4^+^CD25^−^ T cells (*P*<0.01). Two BM samples (patients 8 and 11) and one LN sample (patient 24) were tested, and a representative experiment of three was presented. (B) CD4^+^CD25^−^ cells isolated from a HV or patient with B-cell NHL were co-cultured with purified respective autologous B cells for 5 days. Then, the cells were stimulated with PMA plus ionomycin for 5 hours in the presence of BFA. Cells were harvested and costained with anti-CD3 and anti-CD8. Next, cells were fixed, permeabilized and stained with anti-FoxP3, anti-IL-2, anti-IFN-γ, anti-IL-4, or anti-IL-17. Dot plots were gated on CD3^+^CD4^+^(CD3^+^CD8^−^) T cells, and the numbers within each quadrant represent the percentages of CD4^+^ T cells. Two BM samples (patients 8 and 11) and one LN sample (patient 24) were tested, and the results were representatives of three independent experiments.

Next, we investigated whether the converted Treg acquired the regulatory function. As shown in Figure 5A, the converted Treg could inhibit the proliferation of conventional CD4^+^CD25^−^ T cells. Moreover, the converted FoxP3^+^ cells produced the background level of IL-2, IFN-γ, IL-4 and IL-17, and the FoxP3^−^ cells produced relatively high levels of IL-2, IFN-γ, IL-4 and IL-17 (Figure 5B). The two results suggest that the converted FoxP3^+^ cells be endowed with the classical suppressive function.

## Discussion

Treg can be recruited, expanded or converted in the tumor microenvironment. However, little is currently known about the cause of strong peripheral tolerance in patients with B-cell NHL. In this study, we demonstrated an elevated frequency of CD4^+^CD25^+^FoxP3^+^CD127^lo^ Treg in PB, BM and involved lymphatic tissues from patients with B-cell NHL, and we also showed that these cells could be potently induced by lymphoma B cells in vitro.

We found a significantly higher percentage of CD4^+^CD25^+^FoxP3^+^CD127^lo^ Treg presented in PB as well as BM samples from B-cell NHL compared with PB samples from HVs. Furthermore, the frequencies of CD4^+^CD25^+^FoxP3^+^CD127^lo^ Treg in involved lymphatic tissues were higher than those in PB and BM samples from the same patients. The consequence of these overrepresented CD4^+^CD25^+^FoxP3^+^CD127^lo^ Treg in B-cell NHL remains unknown. In solid tumors, it has been suggested that CD4^+^CD25^+^ Treg are crucial cellular mediators in immune evasion by suppressing anti-tumor immunity and thereby contribute to the growth of human tumors [14], [15], [17]. However, the high numbers of intratumoral FoxP3^+^ Treg were found to be correlated with improved survival in germinal center-like DLBCL, follicular lymphoma and classical Hodgkin's lymphoma [29], [30], [31]. In this study, we have shown that the Treg frequencies in PB were higher in aggressive B-cell NHL than in indolent B-cell NHL. However, in involved lymphatic tissues, there were no significant difference in aggressive B-cell NHL and indolent B-cell NHL, indicating that the enrichment of Treg in involved lymphatic tissues were not associated with the lymphoid tissue types. We thought that malignant B cells might invade PB in aggressive B-cell NHL, which led to an increase in Treg frequency in PB.

We provided direct evidence that CD4^+^CD25^+^ cells in involved lymphatic tissues displayed a higher ability to suppress the proliferation of conventional CD4^+^CD25^−^ T cells than those in PB or BM from patients as well as HVs. Furthermore, we have shown that these Treg were unable to produce and secrete T helper cytokines, suggesting that these cells accord to the classic cytokine profile of Treg [32]. It has been observed that the percentage of CD4^+^FoxP3^+^ cells increased in the co-culture of conventional T cells with malignant B cells, suggesting that malignant B cells can convert conventional CD4^+^CD25^−^ T cells into Treg, which confirm and extend the observations by other investigators [18], [28], [33]. Meanwhile, we have also demonstrated that normal B cells did not have the ability to convert Treg under our experimental conditions. In addition, we have shown that these converted Treg were unable to produce T helper cytokines and could markedly inhibit the proliferation of allogeneic CD4^+^CD25^−^ T cells, which indicated that these converted Treg had acquired the regulatory function. Combined with two previous studies [34], [35], which showed that gastric or ovarian cancer cells could induce the conversion of CD4^+^CD25^−^ T cells into CD4^+^FoxP3^+^ Treg in vitro, we thought that most cancer cells might have the ability of the induction of Treg.

Because the suppressive function of Treg required cell-cell contact, molecules expressed on the cell surface with suppressive function are likely to play a key role in this process [36], [37]. Our results demonstrated that the expression levels of CTLA-4 and GITR were higher in patients with B-cell NHL than those in HVs, suggesting that CTLA-4 and GITR may be involved in modulating suppressive capacity of Treg in human, which might be one of the reasons of higher suppressive ability of Treg from involved lymphatic tissues. Previous studies have shown that blocking of these two receptors on Treg can abrogate the suppressive function, increase anti-tumor immune responses and cause tumor recession [38], [39]. Interaction between PD-1 and B7-H1 results in inhibition of T cell proliferation and cytokine production, which forms a molecular shield to prevent destruction by cytotoxic T cells and remains a weak proliferation of Treg [40], [41]. We have shown that the expression of PD-1 on CD4^+^CD25^+^ Treg was higher in patients with B-cell NHL than in HVs, which strongly suggested a role for PD-1 and B7-H1 interaction in Treg cell-mediated suppression in B-cell NHL. In involved lymphatic tissues, PD-1 was mainly expressed on the CD4^+^CD25^−^ cells, but not on Treg. Blocking the interaction of PD-1 and B7-H1 could reduce the conversion of Treg induced by malignant B cells in some cases of patients with B-cell NHL, which indicated that the Treg conversion of CD4^+^CD25^−^ T cells might be mediated through the PD-1/B7-H1 pathway.

In summary, we have showed an increased frequency and suppressive activity of CD4^+^CD25^+^FoxP3^+^CD127^lo^ Treg in PB, BM, and involved lymphatic tissues from patients with B-cell NHL, which might play a critical role in suppressing the host immune responses to tumor. Furthermore, we have also showed that malignant B cells possessed the intrinsic properties to induce the conversion of Treg from CD4^+^CD25^−^ T cells, which partly through the PD-1/B7-H1 pathway. Thus, these findings open new clues to study Treg biology related to tumor occurrence and provide novel insights into the design of immunotherapeutic strategies for B-cell NHL.
